# The genetic variability of grapevine Pinot gris virus (GPGV) in Australia

**DOI:** 10.1186/s12985-023-02171-3

**Published:** 2023-09-13

**Authors:** Kamalpreet Kaur, Amy Rinaldo, David Lovelock, Brendan Rodoni, Fiona Constable

**Affiliations:** 1 School of Applied Systems Biology, La Trobe University, Bundoora, VIC Australia; 2 The Australian Wine Research Institute, Adelaide, SA Australia; 3 Agriculture Victoria Research, Department of Energy, Environment and Climate Action, Melbourne, VIC Australia

**Keywords:** GPGV, Metagenomic HTS, Phylogenetic analysis, Recombination, Median joining network, Population genetics

## Abstract

Grapevine Pinot gris virus (GPGV; genus *Trichovirus* in the family *Betaflexiviridae*) was detected in Australia in 2016, but its impact on the production of nursery material and fruit in Australia is still currently unknown. This study investigated the prevalence and genetic diversity of GPGV in Australia. GPGV was detected by reverse transcription-polymerase chain reaction (RT-PCR) in a range of rootstock, table and wine grape varieties from New South Wales, South Australia, and Victoria, with 473/2171 (21.8%) samples found to be infected. Genomes of 32 Australian GPGV isolates were sequenced and many of the isolates shared high nucleotide homology. Phylogenetic and haplotype analyses demonstrated that there were four distinct clades amongst the 32 Australian GPGV isolates and that there were likely to have been at least five separate introductions of the virus into Australia. Recombination and haplotype analysis indicate the emergence of new GPGV strains after introduction into Australia. When compared with 168 overseas GPGV isolates, the analyses suggest that the most likely origin of Australian GPGV isolates is from Europe. There was no correlation between specific GPGV genotypes and symptoms such as leaf mottling, leaf deformation, and shoot stunting, which were observed in some vineyards, and the virus was frequently found in symptomless grapevines.

**Supplementary Information:**

The online version contains supplementary material available at 10.1186/s12985-023-02171-3.

## Introduction

*Grapevine Pinot gris virus* (GPGV) is a species in the genus *Trichovirus*, in the family *Betaflexiviridae*. The GPGV genome is a linear, positive-sense, single-stranded RNA of approximately 7259 nucleotides (nt), excluding the poly (A) tail at the 3′ end [1]. It has a typical *Trichovirus* genome with non-coding regions at the 5′ and 3′ ends and three overlapping open reading frames (ORFs): ORF 1 encodes 214 kiloDalton (kDa) virus replicase-associated proteins (1865 aa) including methyltransferase (44–333 aa), helicase (1040–1277 aa) and RNA-dependent RNA polymerase (RdRp) (1447–1797 aa); ORF2 encodes the 42 kDa cell-to-cell movement protein (MP) (367 aa); and ORF3 encodes a 22 kDa viral coat protein (CP) (195 aa) [1]. Previous studies indicate that GPGV is genetically diverse [2–8].

GPGV was first identified in plants of cv. Pinot gris in vineyards of northern Italy in 2012 [1] with characteristic grapevine leaf mottling and deformation disease (GLMD). Other symptoms associated with infection also include delayed budburst, shortened shoot internodes and increased berry acidity [1, 9, 10]. GLMD is caused by GPGV, and infection can lead to serious agronomic losses in sensitive grapevine varieties associated with reduced yield and low quality [11–15]. Since it was first described in Pinot gris, GPGV has been found in other grapevine varieties with GLMD and in varieties that are asymptomatic [1, 2, 6, 16]. Some studies suggest an association between GLMD and specific GPGV strains [1, 4, 6, 17], and one study showed that virus titer and small interfering RNA accumulation were affected by polymorphisms at the 3'end of the movement protein (MP) gene leading to differences in symptom severity [18]. Although significant progress has been made in the understanding of the interaction between GPGV and GLMD disease [15, 19–21], the effects of the GPGV infection on grapevines are still poorly understood, including the relationship between GPGV infection and disease symptoms. Interestingly asymptomatic GPGV infections have been reported in some sensitive varieties that are usually symptomatic, such as Pinot gris and Traminer [2, 22], which cast doubt about its association with GLMD.

It was predicted that GPGV originated from Asia, with China being the most probable source of emergence [5]. Thereafter, GPGV has gradually spread to several grape-producing regions of the world including Europe, USA, Canada, Middle East, and Asia [23]. GPGV colonizes the vascular tissues of grapevines [24] and its global spread is likely due to the movement of infected planting material. Transmission by *Colomerus vitis*, commonly known as grape leaf bud-blister mites, results in the spread of the virus within vineyards [6, 10, 25].

In 2016, GPGV was detected in Australia, New South Wales (NSW), and was subsequently found in Victoria (VIC) and South Australia (SA) [23, 26]. It is suspected that GPGV was introduced to Australia via infected propagation material sometime between 2003 when the movement of the virus into Europe was predicted, and 2014, when testing in Australian post-entry quarantine was introduced [1, 23]. In Australia, GPGV has been found in a broad range of wine grape, table grape and rootstock varieties, but the characteristic GLMD symptoms caused by GPGV infection have not been reported [1, 23]. There has been some recent conjecture that GPGV is associated with a restricted spring growth symptom in Australian table grapes, which includes delayed bud burst, shortened internodes, stunting and zig-zag shoots [23]. To better understand the potential risk of GPGV in Australian vineyards, molecular methods were used to determine the GPGV diversity in rootstock, table, and wine grape varieties, which showed a range of symptoms or were healthy.

## Methods

### Sampling of the grapevine samples

During 2017–2021, Agriculture Victoria’s Crop Health Services (CHS) plant diagnostic laboratory received a total of 2171 samples for GPGV testing from different grape-growing regions of Australia, including 1531 samples from South-eastern Australia in the states of VIC, NSW, and SA. The RNA of 191 GPGV positive CHS samples were selected and retained for further analysisand an additional 126 grapevine samples were collected from southeast Australia for this study (n = 317). Where possible, each of the 317 samples were checked for virus-like symptoms. The 317 grapevines included 70 table grapes, 126 wine grapes and 16 rootstocks. There were 105 grapevines for which the type was unknown.

### RNA extraction and reverse transcription polymerase chain reaction (RT-PCR)

RNA was extracted from 0.3g tissue (fresh weight) of each grapevine sample using the RNeasy® Plant Mini Kit (Qiagen) and eluted in 30μl RNase-free water, as described by Constable et al*.* [27] and quantified using a spectrophotometer (Nanodrop, Thermo Fisher Scientific). Each RNA extract was stored at − 20 °C until use. An RT-PCR assay for the detection of NADH dehydrogenase ND2 subunit (*ndhB* gene, NAD) messenger ribonucleic acid (mRNA) by RT-PCR [28] was used to determine the presence and quality of the extracted RNA.

Each sample was screened using an endpoint RT-PCR assay [4] and a real-time RT-qPCR assay [29]. A GoTaq® 1-Step RT-PCR and RT-qPCR System (Promega) was used according to the manufacturer’s instructions except that the total reaction volume was 25 μl and contained 2 µl of RNA template. The 303bp endpoint RT-PCR amplicons were analyzed by electrophoresis in 2% agarose gels that were stained with SYBR® Safe DNA gel stain (Invitrogen) for visualization. The presence of amplicons corresponding to the size of the genome region of interest was observed on a GelDoc Go Gel Imaging System (Bio-Rad).

### Metagenomic high-throughput sequencing (HTS) library preparation and sequence reads analysis

Thirty-two GPGV-positive grapevines were randomly selected for metagenomic sequencing (Table 1). Five µl of each of the 32 grapevine RNA extracts were used for HTS. The HTS libraries for each sample were prepared using TruSeq® Stranded Total RNA Library Prep Plant with Ribo zero plant kit (Illumina), following the manufacturer’s instructions, and adapters (Perkin Elmer) were used. The size range and concentration of the libraries were determined using the 2200 TapeStation® system (Agilent Technologies) and Qubit® Fluorometer 2.0 (Invitrogen), respectively, and the resulting quantification values were used to pool the libraries. The resulting library was finally sequenced using the NovaSeq 6000 system (Illumina) with a paired read length of 2 × 150 bp.

**Table 1:** The metadata for each of the thirty-two grapevine Pinot gris virus-infected grapevines that were selected for high throughput sequencing (HTS) in this study including the year collected, grapevine type, variety, geographic origin, and symptoms observed

Sample id	Year collected	Rootstock, wine or table grape	Variety	Location	State	Symptoms
CK1	2017	Rootstock	101–14	Mildura^a^	VIC	Asymptomatic
2.1	2019	Table	Ralli seedless	Mildura	VIC	Stunted growth
2.12	2019	Table	Adora	Robinvale	VIC	Asymptomatic
2.17	2019	Table	Sweet Angie	Euston	NSW	Stunted growth
5.5	2020	Table	Crimson seedless	Mildura	VIC	Stunted growth, zig-zag shoots, shorter nodes, small bunches of fruit
5.6	2020	Table	Unknown	Mildura	VIC	Asymptomatic
5.13	2020	Wine	Fresno seedless	Mildura	VIC	Stunted growth bud-blister mites, tighter fruit
5.14	2020	Wine	Pinot gris	Mildura	VIC	Asymptomatic
5.17	2020	Table	Ralli seedless	Mildura	VIC	Stunted growth, zig-zag shoots, clump together leaves, cabbagy leaves
5.21	2020	Table	Ralli seedless	Mildura	VIC	Stunted growth, short internodes, zig-zag shoots
5.22	2020	Table	Ralli seedless	Mildura	VIC	Stunted growth, short internodes, zig-zag shoots
5.24	2020	Table	Sugar crisp	Mildura	VIC	Stunted growth
8.6	2021	Wine	Unknown	Angaston	SA	Asymptomatic
8.7	2021	Wine	Unknown	Angaston	SA	Asymptomatic
8.28	2021	Wine	Ansonica	Mildura	VIC	Asymptomatic
8.29	2021	Wine	Ansonica	Mildura	VIC	Asymptomatic
8.33	2021	Wine	Lambrusco	Mildura	VIC	Asymptomatic
8.38	2021	Wine	Nero d'Avola	Mildura	VIC	Asymptomatic
8.47	2021	Wine	Vermentino	Mildura	VIC	Asymptomatic
LT6	2021	Wine	Grüner Veltliner	Adelaide Hills	SA	Asymptomatic
LT7	2021	Wine	Grüner Veltliner	Adelaide Hills	SA	Asymptomatic
9.1	2021	Table	Ralli seedless	Mildura	VIC	Faint mottling on leaves, streaking on fruit, secondary bud dries off, tight fruit, stunted growth
9.2	2021	Table	Ralli seedless	Mildura	VIC	Faint mottling on leaves, streaking on fruit, secondary bud dries off, tight fruit, stunted growth
9.3	2021	Table	Ralli seedless	Mildura	VIC	Faint mottling, streaking on leaves, zigzag shoots, stunted growth
9.4	2021	Table	Ralli seedless	Mildura	VIC	Faint mottling, streaking on leaves, zigzag shoots, stunted growth
9.5	2021	Wine	Nero d'Avola	Mildura	VIC	Leaf mottling and deformation, restricted spring growth, stunted growth
9.6	2021	Wine	Fiano	Mildura	VIC	Leaf mottling and deformation, restricted spring growth, stunted growth
9.9	2021	Wine	Malbec	Mildura	VIC	Leaf mottling and deformation, restricted spring growth, stunted growth
9.10	2021	Wine	Vermentino	Mildura	VIC	Leaf mottling and deformation, restricted spring growth, stunted growth
9.11	2021	Table	Crimson seedless	Mildura	VIC	Leaf mottling and deformation, restricted spring growth, stunted growth
9.13	2021	Wine	Vermentino from a field nursery	Mildura	VIC	Zig-zag shoots, restricted spring growth, short internodes, stunted growth
9.14	2021	Wine	Malbec from a field nursery	Mildura	VIC	Zig-zag shoots, restricted spring growth, small bunches, no fruits, 2nd year is zigzag, stunted growth

^a^Mildura refers to the Victorian area of the Sunraysia horticultural region surrounding the city of Mildura and extends 60 km from Yelta in the northwest and Colignan in the southeast

### Bioinformatics analysis

All the raw data was quality filtered, adapters were trimmed, and the generated sequence read pairs were validated using Fastp (version 0.20.0) with default parameters. De novo assembly of the quality-checked paired sequence reads into contigs was carried out using the genome assembler SPAdes (version 3.13.0) [30]. The resulting de novo assembled contigs were searched [31] against the NCBI nucleotide database using the alignment search tool BLASTn for the presence of GPGV and other viruses in the grapevine samples. Reference mapping of the de novo assembled contigs for each sample was done using Bowtie2 (version 2.3.4.2) using the most similar genome identified in the previous BLASTn search after which the mapped consensus sequence was viewed in Geneious (version 11.0) to determine the mapped reads coverage and average depth of the genomes generated for each sample.

### RT-PCR confirmation of viruses detected by high-throughput sequencing (HTS)

The arrangement of the coding region (equivalent to nt positions 22 to 6812 of the reference isolate NC_015782) of the assembled genomes of two GPGV isolates (5.21 and LT6) that were generated by HTS was confirmed by Sanger sequencing of overlapping amplicons, to assure their quality. The overlapping amplicons were generated by RT-PCR with primer pairs that were designed in this study using Oligo Explorer (version 1.1.2; www.genelink.com/tools/gl-oe.asp) and three published primer pairs (Additional file 1: Table S1) [4, 6, 32]. PCR amplification and gel electrophoresis were done as described previously. The amplicons were purified using QIAquick® PCR & Gel Cleanup Kit (Qiagen) and sent to Macrogen (Seoul, Korea) for Sanger sequencing. Each amplicon was sequenced twice in the forward and reverse directions. The resulting sequences for each isolate were used in combination with the contigs assembled from the HTS data to generate consensus genome sequences for each isolate. Four GPGV isolates (5.5, 5.13, 5.24 and LT7) had genomes with low average coverage and depth and in some cases, gaps in the consensus, therefore Sanger sequencing of specific regions were used to complete and/or confirm the genome assembly.

### Phylogenetic tree and sequence identity analyses

To establish a relationship between the manifestation of symptoms and specific strains of GPGV, phylogenetic analysis of a 460nt region of the GPGV genome encompassing the 3′ end of the movement protein and the 5′ end of the coat protein ORFs [2, 6] of 32 Australian isolates was compared to representative isolates previously described as associated with GLMD symptoms or with asymptomatic infections [6, 17]. Multiple alignments were performed using the MEGA X software [33] with default parameters. Phylogenetic trees were generated using the maximum likelihood (ML) method based on the Tamura-Nei model with 1000 bootstrap replicates.

The consensus genome sequences of the 32 Australian GPGV isolates generated in this study were aligned with 168 GPGV genome sequences available in GenBank (Additional file 1: Table S2) using MUSCLE alignment software [34], excluding the viral untranslated regions (UTRs). The genetic distances within the isolate groups were calculated and maximum-likelihood phylogenetic trees were constructed using Kimura’s two parameter model in MEGA X [33] with default parameters and 1000 bootstrap replicates. The sequence identity analysis was carried out using BioEdit Sequence Alignment Editor [35] and the Sequence Demarcation Tool (version 1.2) [36] on the aligned genome sequences. The sequence similarity percentages of the isolates were determined at the nucleotide level for GPGV by MUSCLE alignment implemented in the SDT software (version 1.2).

Based on current taxonomic demarcation criteria recommended by the International Committee on Taxonomy of Viruses (ICTV) for the *Betaflexiviridae* [37, 38], phylogenetic trees for the RdRp and CP region of GPGV were constructed and the sequence similarity percentage was determined at both the nucleotide and amino acid (aa) levels using the methods mentioned above.

### Median-joining (MJ) network, population genetics, equilibrium model, neutrality test and fixation index analyses

Variants networks were created using the Median Joining (MJ) algorithm and visualized using the PopART software (http://popart.otago.ac.nz), with default settings, for 51 aligned GPGV genome sequences including 32 Australian isolates and 19 overseas isolates that were used in the phylogenetic analysis and had highest nucleotide identity with the Australian isolates.

Further, the aligned genome sequences of the 32 GPGV isolates generated in this study along with 168 overseas GPGV isolates available in GenBank were also used to assess the genetic differentiation parameters such as number of variants (V), variants diversity (Vd), number of polymorphic (segregation) sites (S), the total number of mutations *η* (Eta), the average number of nucleotide differences (k) and average pairwise nucleotide diversity (π), the total number of synonymous sites (SS), the total number of non-synonymous sites (NS) and the ratio of non-synonymous nucleotide diversity to synonymous nucleotide diversity ($\omega = dN{/}dS)$ using the DnaSP software (version 6.10.01) [39].

The *Tajima’s D* [40] statistical test of neutrality, included in the DnaSP software, was also used on the dataset with default sliding window parameters, to test the neutral selection hypothesis on the GPGV genomes between populations. This is to determine whether the viral populations are evolving under a non-random process (*D*_*T*_ > 0: balancing selection, sudden population decline); mutation-drift equilibrium (*D*_*T*_ = 0) or a recent selective sweep (*D*_*T*_ < 0: population expansion after a recent bottleneck). The coefficient of *F*_*ST*_ (fixation index) is a measure of the average pairwise distances between pairs of individual variants in terms of allele frequencies and was calculated by performing 1000 sequence permutations in DnaSP to estimate the genetic differentiation between populations. The fixation index (F_ST_) can range from 0 to 1, where 0 indicates no differentiation between populations and 1 indicates populations are completely isolated and there is no sharing of genetic material or gene flow [39, 41, 42].

### Recombination analysis

The aligned genome sequences of virus isolates from this study and the sequences of corresponding GPGV isolates available in GenBank were checked for potential recombination events. When screening for recombination, likely parental isolates of potential recombinants and recombination breakpoints within the genome sequences of GPGV from this study were determined using the RDP program (version 4.9) [43] with default parameters [44]. A recombination event was considered to be genuine only if it was detected by four or more of the seven measures [RDP (R), GENECONV (G), BOOTSCAN (B), MAXCHI (M), CHIMAERA (C), SISCAN (S), and 3SEQ (Q)] with *p* values < 0.05, implemented in the software RDP4.9 [45–47]. Recombination signals were disregarded if they were flagged by RDP4.9 as potentially arising through evolutionary processes other than recombination.

## Results

### RT-PCR results

**Fig. 1:**
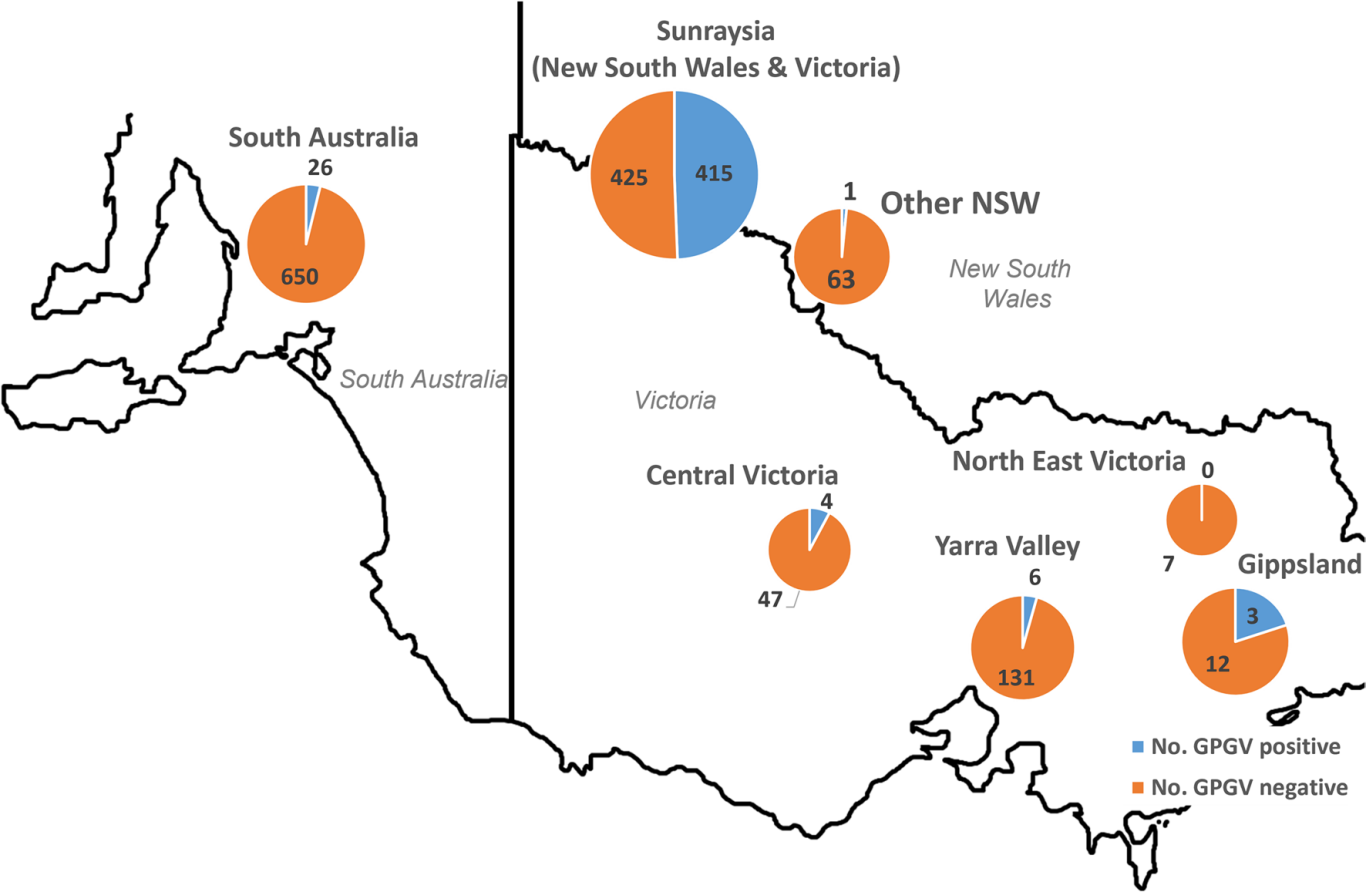
The number of samples submitted to Crop Health Services Plant Diagnostic Laboratory from various grape growing regions in South Australia (SA), New South Wales (NSW) and Victoria (VIC) that tested positive or negative for grapevine Pinot gris virus (GPGV). The Sunraysia horticultural district encompasses growers located in northwest VIC and southwest NSW. Other NSW isolates in the image includes samples from various regions in that state. Image generated by tracing Google Maps (Scale = 20 km)

GPGV was detected in 473/2171 (21.8%) of the samples tested, including 23/133 samples from NSW, 26/706 samples from SA and 424/1176 samples from VIC. GPGV was not detected in 23 samples from Tasmania or 133 samples from Western Australia. The samples from VIC and NSW were analyzed by region (Fig. 1) and the highest proportion of positive samples (415/840; 49.4%) was observed in the Sunraysia horticultural district which encompasses northwestern VIC (350 samples) and southwestern NSW (70 samples).

Using retained RNA of the CHS samples and samples collected specifically for this study (n = 317), GPGV was detected in 113/317 samples, including 37/70 table grape, 59/126 wine grape, 2/16 rootstock and 15/105 unknown varieties based on the presence of the expected 303bp amplified PCR product [4] as well as Ct values ranging from 16.24 to 35.24 (data not shown) [29]. The presence of GPGV was confirmed in 81/191 reanalyzed CHS RNA extracts. Most of the GPGV-positive samples (101) were from the Sunraysia region in VIC and surrounding areas, with the remaining samples from the Sunraysia region in NSW (9) and vineyards in SA (3).

**Fig. 2:**
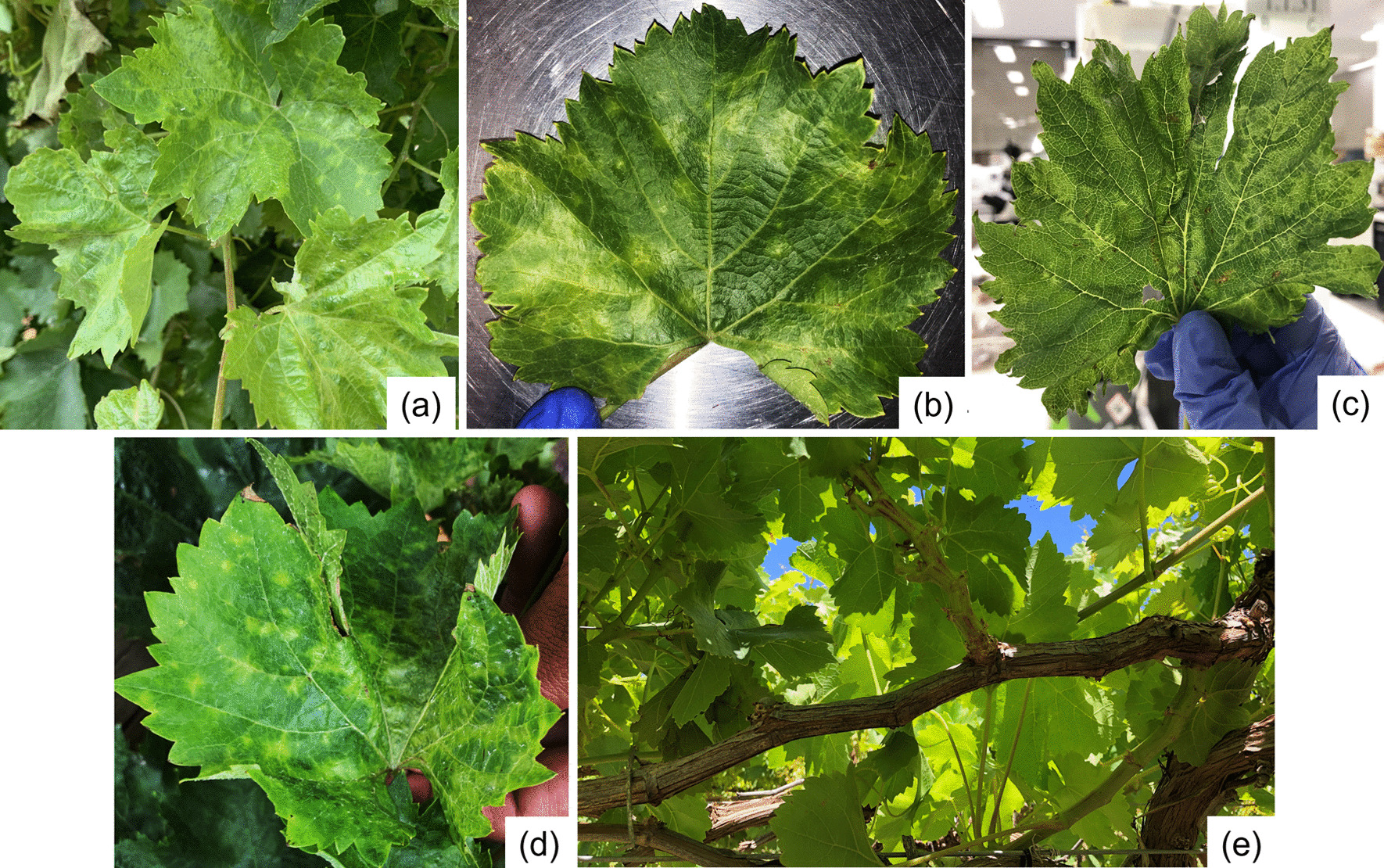
Symptoms were observed in different table and wine grape varieties. Leaf mottling and deformation symptoms were observed in (**a**) Nero d’Avola; (**b**) Fiano; (**c**) Vermintino; (**d**) Malbec and zig-zag shoots observed in (**e**) Crimson seedless

Symptoms were not recorded for 14 GPGV-positive grapevines, which were submitted through the CHS plant diagnostic laboratory for virus testing. Of the remaining 99 GPGV-positive grapevines, 6 had GLMD-like symptoms, 31 grapevines had a range of symptoms including restricted spring growth, millerandage and zigzag shoots and 62 were asymptomatic (Fig. 2).

### Metagenomic high-throughput sequencing (HTS) and bioinformatics analysis

The total raw reads generated by metagenomic HTS from 32 grapevine samples ranged from 81,949 to 30,198,881 reads/sample and these numbers were reduced to 81,714 to 29,489,728 reads/sample after quality trimming. De novo assembly of reads from each sample using SPAdes resulted in 3329–178,375 contigs from which 10 to 2674 contigs matched with known viral sequences. Of those virus-related contigs, 1–12 contigs matched most closely to GPGV across the 32 samples which were confirmed to be GPGV after a BLASTn [31] search of the GenBank database. The genome size of the various GPGV contigs in the different samples ranged from 6574 to 7449 nt with the average size of contigs per GPGV genome ranging from 1534 to 7426 nt (Additional file 1: Table S3). The most complete genome sequences for the GPGV strains found in each grapevine sample were used for downstream analysis.

Sanger sequencing of overlapping amplicons generated by RT-PCR confirmed the genome sequence of the two Australian exemplar isolates 5.21 and LT6 and completed the assembly of the GPGV genome for isolates 5.5, 5.13, 5.24 and LT7, which had gaps and low coverage across the assembled genomes. The consensus genome sequences (excluding UTRs) generated in this study for 32 Australian GPGV isolates are available on GenBank (Accession number: OQ198990-OQ199021).

### Phylogenetic and sequence identity analysis

**Fig. 3:**
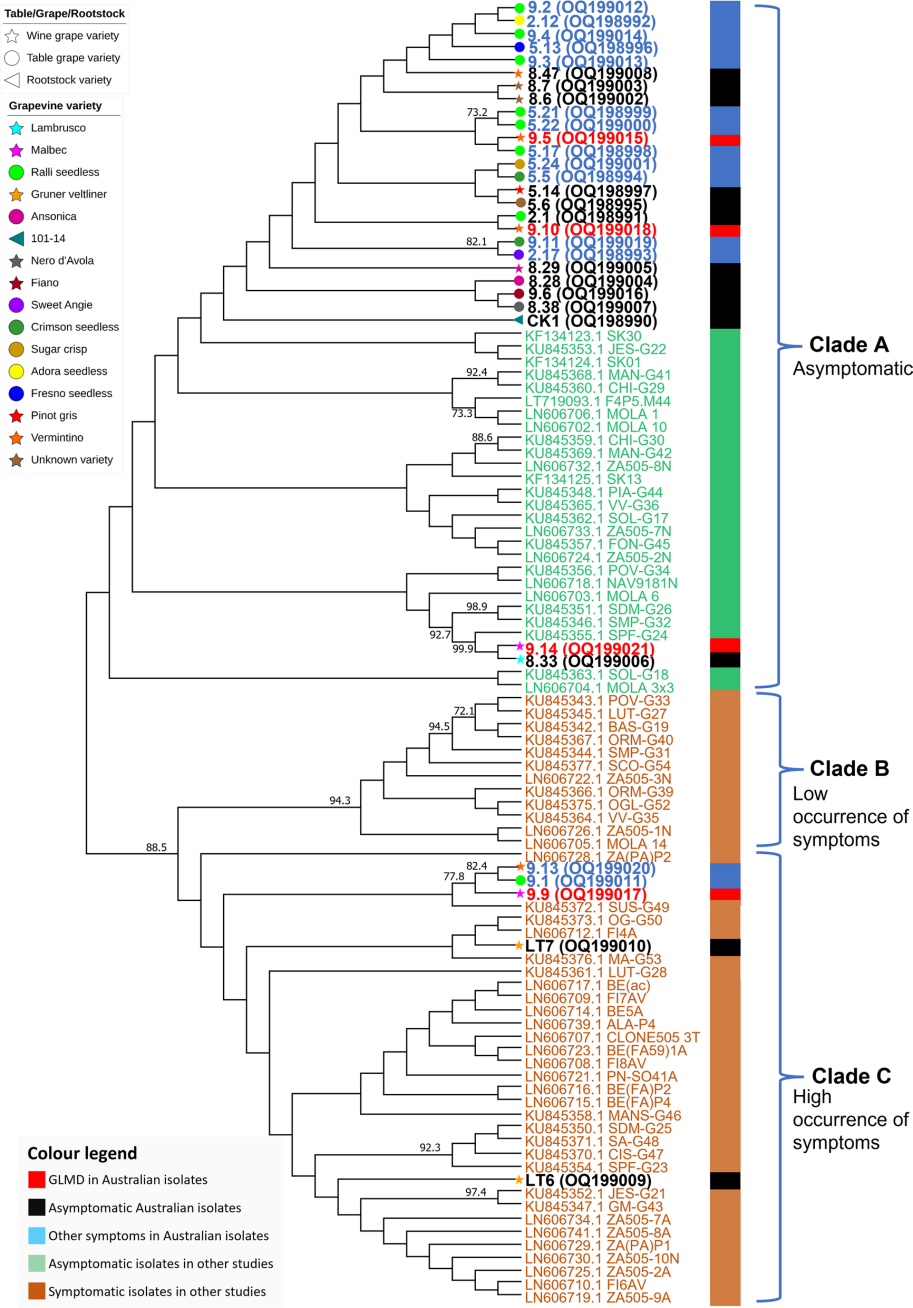
Maximum likelihood phylogenetic tree with 1000 bootstrap replicates of the 3′ end of movement protein and 5′ end of coat protein genes of the genome for 32 Australian grapevine Pinot gris virus (GPGV) isolates and 68 isolates from previous studies that were associated with grapevine leaf mottling and deformation disease (GLMD) or with asymptomatic infections in various grapevine varieties. Clade A (asymptomatic grapevines) and clades B and C (GLMD-affected grapevines) are based on the clades described previously [2, 6]. Bootstrap values (> 70%) are reported at the nodes

The phylogenetic relationships between the 32 GPGV isolates from Australia and representative overseas isolates found on symptomatic and asymptomatic grapevines in previous studies were assessed using a 460nt genomic region encompassing the 3′ end of the MP and 5′ end of the CP ORFs [2, 6]. Five Australian GPGV isolates, including one isolate associated with GLMD-like symptoms (9.9), two isolates from grapevines with other symptoms including restricted spring growth, millerandage and zigzag shoots (9.1, 9.13), and two isolates that were from asymptomatic grapevines (LT6, LT7) were in clade C (high occurrence of symptoms), that included isolates from other countries that were associated with GLMD. The remaining 27 Australian isolates, which included three isolates (9.10, 9.5, 9.14) from grapevines with GLMD-like symptoms, 12 isolates from grapevines with other symptoms and 11 isolates from asymptomatic grapevines, were in clade A that included isolates from other countries that were detected in asymptomatic grapevines (Fig. 3).

**Fig. 4:**
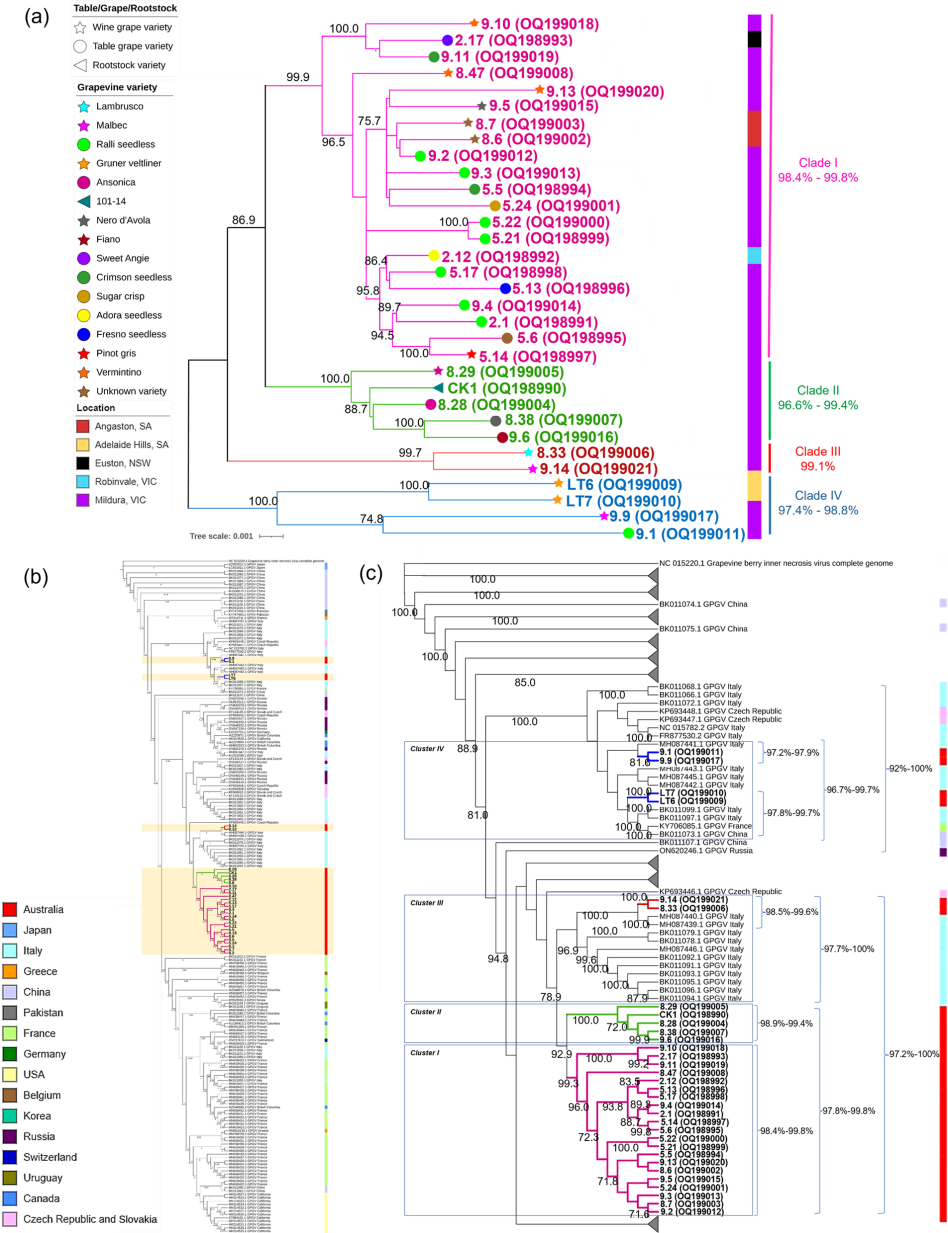
Maximum likelihood tree inferred from thirty-two genome sequences of grapevine Pinot gris virus (GPGV) which were de novo assembled from high throughput sequencing (HTS) datasets (**a**) only Australian isolates; **b** full tree with 32 Australian and 168 overseas isolates published on GenBank; **c** collapsed tree to highlight the global relationships of Australian isolates to isolates from other regions. The number at each node indicates bootstrap percentages based on 1000 replicates. The scale bar corresponds to the number of substitutions per site. The percentage identity to each other for each clade is on the right

**Fig. 5:**
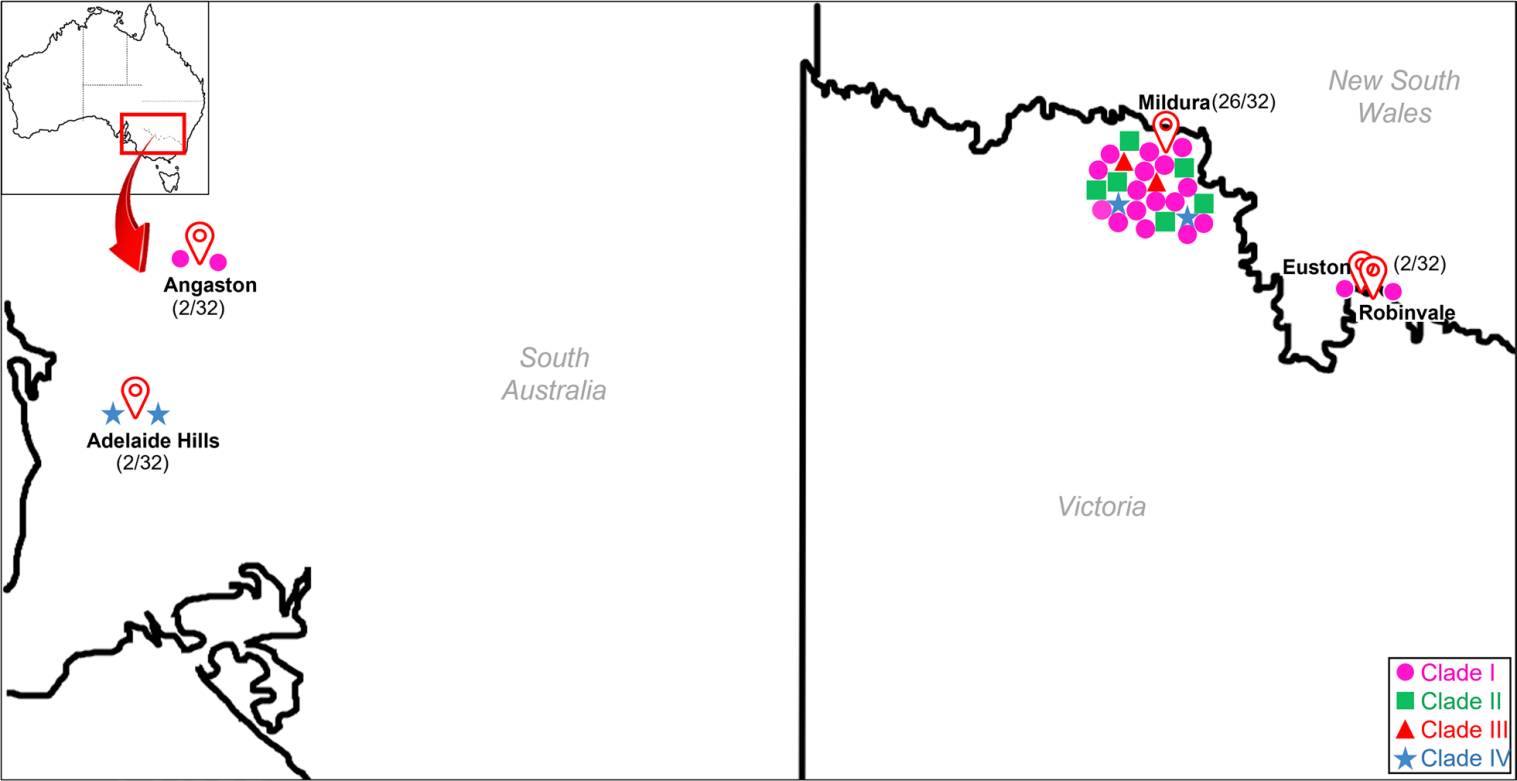
Location of sample collection sites of GPGV isolates in different vine-growing regions of Australia. Image generated by tracing Google Maps (Scale = 20 km). Mildura refers to the Victorian area of the Sunraysia horticultural region surrounding the city of Mildura and which extends 60km from Yelta in the northwest and Colignan in the southeast

Isolates of all four clades were found in the Sunraysia grape-growing region of VIC (Mildura and surrounding towns, Robinvale) and NSW (Euston) (Figs. 4a, 5). Only clade I and clade III isolates were found in SA, from the Barossa Valley (Angaston) and Adelaide Hills (AH), respectively. The SA clade I isolates, which shared 99.3% nt identity with each other, were from the same Barossa Valley grower, and the SA clade III isolates, which shared 98.8% nt identity with each other, were from the same Adelaide Hills grower.

The nucleotide percentage identity between the Australian GPGV isolates ranged from 96.6 to 99.8% for the RdRp gene and 93.1 to 100% for the CP gene (Additional file 1: Fig. S1). The overall percentage amino acid identity between the Australian GPGV isolates ranged from 97.7 to 100% for the RdRp protein and 93.9 to 100% for the CP gene (Additional file 1: Fig. S2).

The genomes (excluding UTRs) of 32 Australian and 168 overseas GPGV isolates shared 69–100% nucleotide identity with each other. Phylogenetic analysis of the genomes demonstrated that Australian GPGV isolates in Australian clades I and II (defined in Fig. 4a) formed a distinct cluster that was most closely related to a cluster of isolates from Italy, and which also contained the two GPGV isolates from Australian clade III (Fig. 4b, c). Australian clades I and II share 97.8–99.8% nucleotide identity, the Australian clade III and Italian isolates share 97.7–100% nucleotide identity, and the larger cluster of the three Australian clades and the Italian isolates shared 97.2–100% nucleotide identity. Australian clade III isolates which includes isolates 8.33 and 9.14, and which were detected in asymptomatic Lambrusco and Malbec with GLMD-like symptoms respectively, were most closely related (98.5–99.6% nucleotide identity) to isolates fvg-Is6 (MH087440) and fvg-Is1 (MH087439), which were isolated from asymptomatic grapevines.

Australian clade IV GPGV isolates fell into a second distinct cluster of isolates that were primarily from Europe and also included one isolate from China (Fig. 4b, c). These isolates shared 96.7–99.7% nucleotide identity with each other. Australian grapevine isolates 9.1 (asymptomatic Ralli seedless table grape) and 9.9 (Malbec with GLMD-like symptoms) from the Sunraysia region (Mildura), were most closely related to isolate fvg-Is7 from Italy (MH087441), which was isolated from a symptomatic grapevine. Isolates LT6 and LT7, from asymptomatic Grüner Veltliner were most closely related (97.8–99.7% nt identity) to two Cabernet sauvignon isolates from Italy (BK011097, BK011099), one Cabernet sauvignon isolate from China (BK011073) of unknown disease status, and one isolate from a symptomatic grapevine cv. Pinot noir in France (KY706085).

### Median-joining (MJ) network, population genetics, equilibrium model, neutrality test and fixation index analysis

**Fig. 6:**
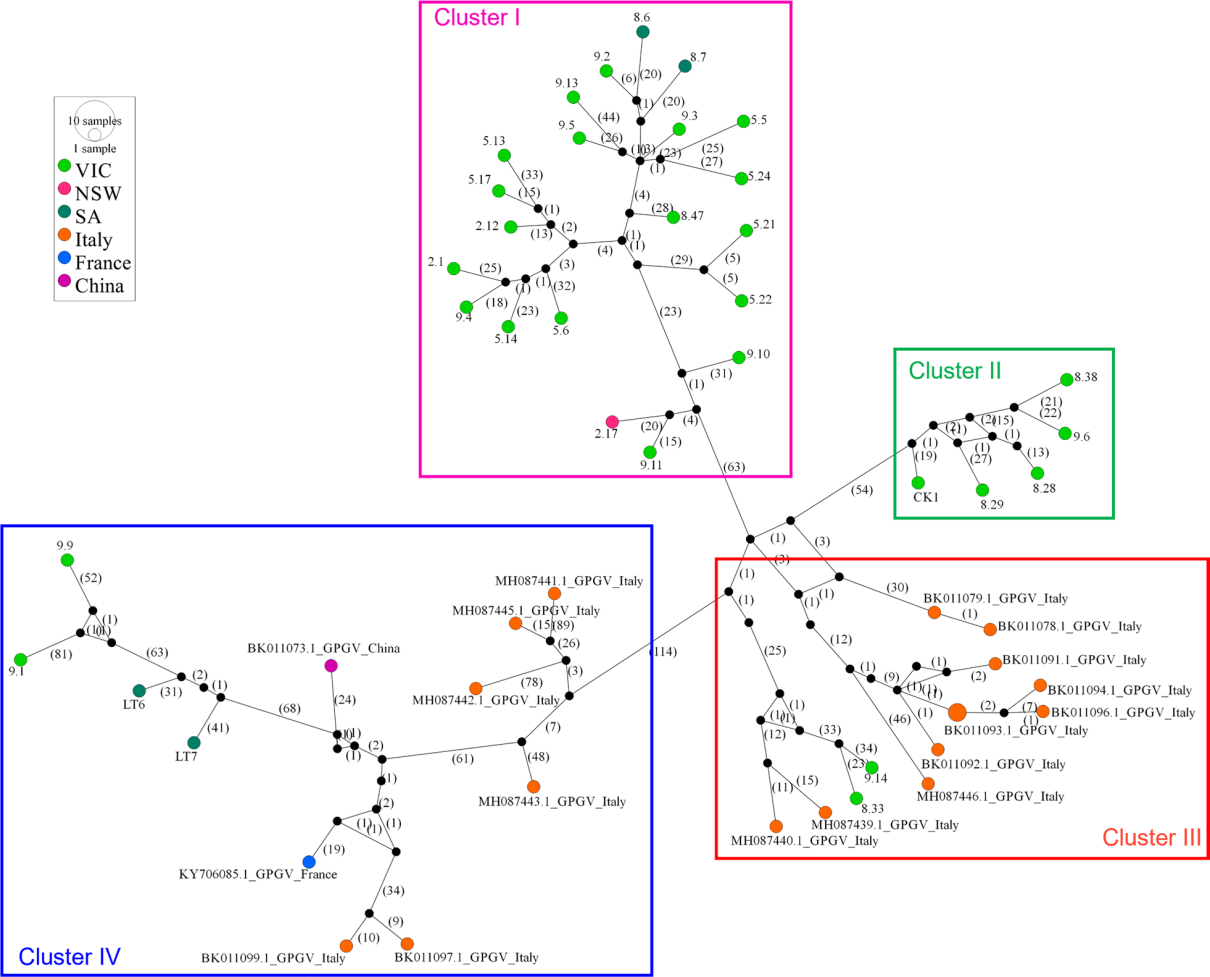
Median-joining network showing grapevine Pinot gris virus (GPGV) variants dividing the isolates into four distinct clusters including 32 GPGV isolates from Australia and 19 overseas isolates. The sizes of the circles are proportional to the number of times each variant was observed. Unlabelled black dots (median vectors) are hypothetical intermediate variants connecting the variant groups. Numbers in the bracket infer the number of mutations separating the variants

MJ networks of 32 GPGV isolates from Australia and 19 overseas isolates, which clustered with Australian isolates in the Australian clades I-IV of the phylogenetic tree (Fig. 4c), showed four distinct variant clusters (Fig. 6). The formation of these clusters is supported by the phylogenetic analysis in which four clades were formed that contained the same Australian and overseas isolates (Fig. 4c) as the related median-joining network clusters (Fig. 6). Each MJ network cluster contained hypothetical intermediate variants (represented by black dots) that were often directly linked to only one or a few of the known variants that were analyzed.

Genetic diversity parameters and selective pressure were analyzed for the 32 Australian and 168 overseas GPGV isolates (Table 2). The sequences were grouped and analyzed based on clusters formed in the phylogenetic analysis (Fig. 4c). All four populations share a high level of variant diversity (Vd) which is either 1 or closer to one, indicating high levels of diversity for each cluster. Low nucleotide diversity (π) was observed and ranged between 0.008 and 0.022 within the four MJ network clusters indicating GPGV population expansions (Table 2). The population of GPGV isolates in the four clusters had a ratio of < 1 for nonsynonymous nucleotide diversity to synonymous nucleotide diversity (ω) indicating that they have been under a purifying selection (Table 2). The neutrality test (*Tajima’s Dt*) produced negative values for each cluster and are indicative of low population differentiation and infer population growth (Table 2). The fixation index (F_ST_) test statistic, which was used to estimate the degree of genetic divergence between the MJ network clusters, ranged between 0.44 and 0.52 for each pair of clusters that were compared, suggesting infrequent gene flow and high genetic differentiation (Table 3).

**Table 2:** Summary of genetic variation and neutrality test analysis of grapevine Pinot gris virus (GPGV) genomes for different populations formed by four phylogenetic clusters which include 32 Australian and 19 overseas GPGV isolates from France, Italy, and China

Phylogroup	N	V	Vd	S	η	k	π	SS	NS	ω	D_T_	*P* value
Cluster 1	21	21	1.000	463	467	62.26	0.010	1398	5211	0.262	− 2.154	*P* < 0.01
Cluster 2	5	5	1.000	117	117	51.40	0.008	1402	5213	0.284	− 0.645	*P* > 0.10
Cluster 3	12	11	0.985	264	266	69.72	0.011	1405	5210	0.294	− 0.859	Not significant, *P* > 0.10
Cluster 4	12	12	1.000	578	584	142.8	0.022	1398	5199	0.245	− 1.230	Not significant, *P* > 0.10

N, number of sequences; V, number of variants; Vd, variant diversity; S, number of polymorphic (Segregating) sites; η, the total number of mutations; k, the average number of nucleotide differences between sequences; π, nucleotide diversity; SS, the total number of synonymous sites analyzed; NS, the total number of non-synonymous sites analyzed; Pi(s), synonymous nucleotide diversity; Pi(a), non-synonymous nucleotide diversity; ω = dN/dS; D_T_, Tajima's D_T_ value; P value, statistical significance

**Table 3:** Measurements of population’s differentiation (fixation index, F_ST_) of genome sequences of the four clusters formed phylogenetically with the 32 Australian grapevine Pinot gris virus (GPGV) and 19 representative overseas isolates from France, Italy, and China

Population 1	Population 2	F_ST_
Cluster_1	Cluster_2	0.52
Cluster_1	Cluster_3	0.51
Cluster_1	Cluster_4	0.48
Cluster_2	Cluster_3	0.50
Cluster_2	Cluster_4	0.51
Cluster_3	Cluster_4	0.44

### Recombination analysis

**Fig. 7:**
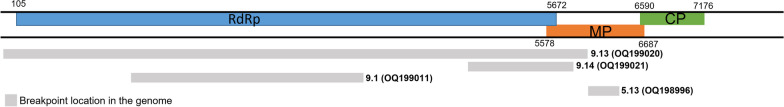
The location of the recombination events on the genome of four Australian grapevine Pinot gris virus (GPGV) isolates as detected by recombination-detection algorithms (RDP4 program)

The analysis of 168 global isolates did not identify any international isolates as major or minor parents of 32 Australian GPGV isolates. However, four statistically significant (*p* < 0.05) recombinants were were predicted by the analysis (Table 4): recombinant 1—9.13 (clade I) with major parent 9.9 (clade IV) with 97.0% similarity and minor parent 5.13 (clade I) with 98.6% similarity; recombinant 2—9.14 (clade III) with major parent 8.33 with 99.1% similarity and minor parent 9.1 (clade IV) with 96.9% similarity; recombinant 3—9.1 (clade IV) with major parent 9.9 (clade IV) and minor parent 8.47 (clade I); recombinant 4—5.13 (clade I) with major parent 2.1 (clade I) with 99.0% similarity (refer to Fig. 4a). Recombination affected the RdRp gene in three outcomes (isolates 9.1, 9.13, 9.14) and the MP gene in one outcome (isolate 5.13) (Fig. 7, Table 4). All recombinants and parents were located in the Sunraysia region of VIC (Mildura), within approximately a 25km radius.

**Table 4:** The number of predicted recombinants and recombination events identified using the RDP4.9 package within the genome sequences of grapevine Pinot gris virus (GPGV) isolates

Recombinant name	Breaking point location^a^		Parents				Genes affected^c^	Programs detected by^d^
	Beginning	End	Major	% Similarity^b^	Minor	% Similarity^b^		
9.13	61	6139	9.9	97	5.13	98.6	RdRp, MP	M, C, S, 3
9.14	4853	5942	8.33	99.1	9.1	96.9	RdRp, MP	R, G, B, M, C, S, 3
9.1	1110	3819	9.9	98.0	8.47	97.5	RdRp	R, G, B, M, C, S, 3
5.13	6139	6254	2.1	99.0	Unknown	–	MP	R, G, B, S, 3

^a ^Numbers represent nucleotide position in the GPGV genome

^b ^% Similarity generated using BioEdit

^c ^RdRp, RNA-dependent RNA polymerase; MP, Movement protein; CP, Coat protein

^d ^R, RDP; G, GENECONV; B, Bootscan; M, Maxchi; C, Chimaera; S, SiScan; 3, 3Seq

## Discussion

This study provides a snapshot of the prevalence of GPGV in wine, table grape and rootstock varieties in Australia between the years 2017 and 2021. GPGV was detected in grapevines with GLMD-like symptoms as well as in grapevines with other symptoms such as delayed budburst, increased berry acidity, stunted shoots, poor yield, restricted spring growth, zig-zag shoots and millerandage. The virus was also detected in asymptomatic grapevines. GPGV was only found in southeastern Australia, in NSW, SA and VIC and had the highest prevalence in the Sunraysia horticultural region, where table grapes and wine grapes are grown and where some germplasm collections and grapevine nurseries are located.

There is no doubt that infected planting material is responsible for the introduction of GPGV into Australia, and it has been reported that once testing was introduced at the Australian border, 10% of imported grapevines had GPGV [23]. The close relationship of many Australian isolates to Italian isolates, demonstrated by the phylogenetic analysis, supports the hypothesis that the introduction of GPGV into Australia has a European origin. This is further supported by the MJ variant network analysis, in which European isolates and one Chinese isolate occur with Australian isolates in cluster IV and Italian and Australian isolates occur together in cluster III. It is likely that GPGV was introduced into Australia from Europe after it was introduced into Italy, which is estimated to have occurred in 2003, and before the time testing commenced at the Australian border in 2014 [23, 26].

The Australian and overseas GPGV isolates in clades I and II are closely related suggesting that GPGV isolates from both clades may have emerged from one introduction into Australia (Fig. 4b). However, the MJ network indicates that they are distinct clusters emerging from different hypothetical intermediate variants and therefore it is more likely that phylogenetic clades I and II are associated with separate introductions (Fig. 6). Thus, the presence of four distinct Australian phylogenetic clades and four MJ network clusters suggests at least four different introductions of GPGV into the country. The presence of GPGV isolates from Sunraysia in all four clades and MJ network clusters suggests multiple introductions of the virus into this region. Isolates LT6 and LT7 from SA, which are closely related to Sunraysia GPGV isolates found in Malbec (9.9) and Ralli Seedless (9.1), were derived from Gruner veltliner planting material that was imported independently from Europe and was not distributed to or in contact with material from Sunraysia at the time this study was conducted. This indicates a minimum of five introductions of GPGV into Australia. However, it is possible that these four closely related isolates have a common European origin.

RNA viruses are known to have high mutation rates which lead to the production of deleterious mutations that can destabilize the virus population [48, 49]. Thus, when RNA viruses with a large population size reach a bottleneck, a purifying selection helps in eliminating these mutants and improves the survival of the population [48, 49]. The neutrality test parameter *D*_*T*_ is known to test the distribution of nucleotide polymorphisms in the genome [40]. The negative numbers of *D*_*T*_ suggest a recent introduction of this virus in Australia and infer a recent expansion of Australian GPGV populations through new mutations. Virus populations were shown to survive bottleneck selections, which were indicated by negative neutrality test values, and could be caused by a transmission during grafting or transmission by the vector bud-blister mites. The presence of hypothetical intermediate variants in the MJ network clusters is an indicator of diversity within the clusters. Further sampling and sequencing of GPGV isolates is required to determine how the known variants are linked to a specific variant introduced into Australia or if multiple introductions of closely related variants has occurred.

The *F*_*ST*_ values of 0.44–0.52 between the four clusters suggest that the clusters are linked. This is likely due to the high relatedness of the Italian isolates that are linked to the five introductions of GPGV into Australia. There is some association between Australian isolates in cluster IV and an isolate from China (BK011073) and therefore an introduction from this region cannot be ruled out. However, Australia has imported most grapevine material from Europe or the Americas and an introduction from Europe seems more likely. It is also possible that the Chinese isolate, which was detected in the variety Cabernet sauvignon, is also a result of the importation of infected grapevine material from Europe into China.

**Fig. 8:**
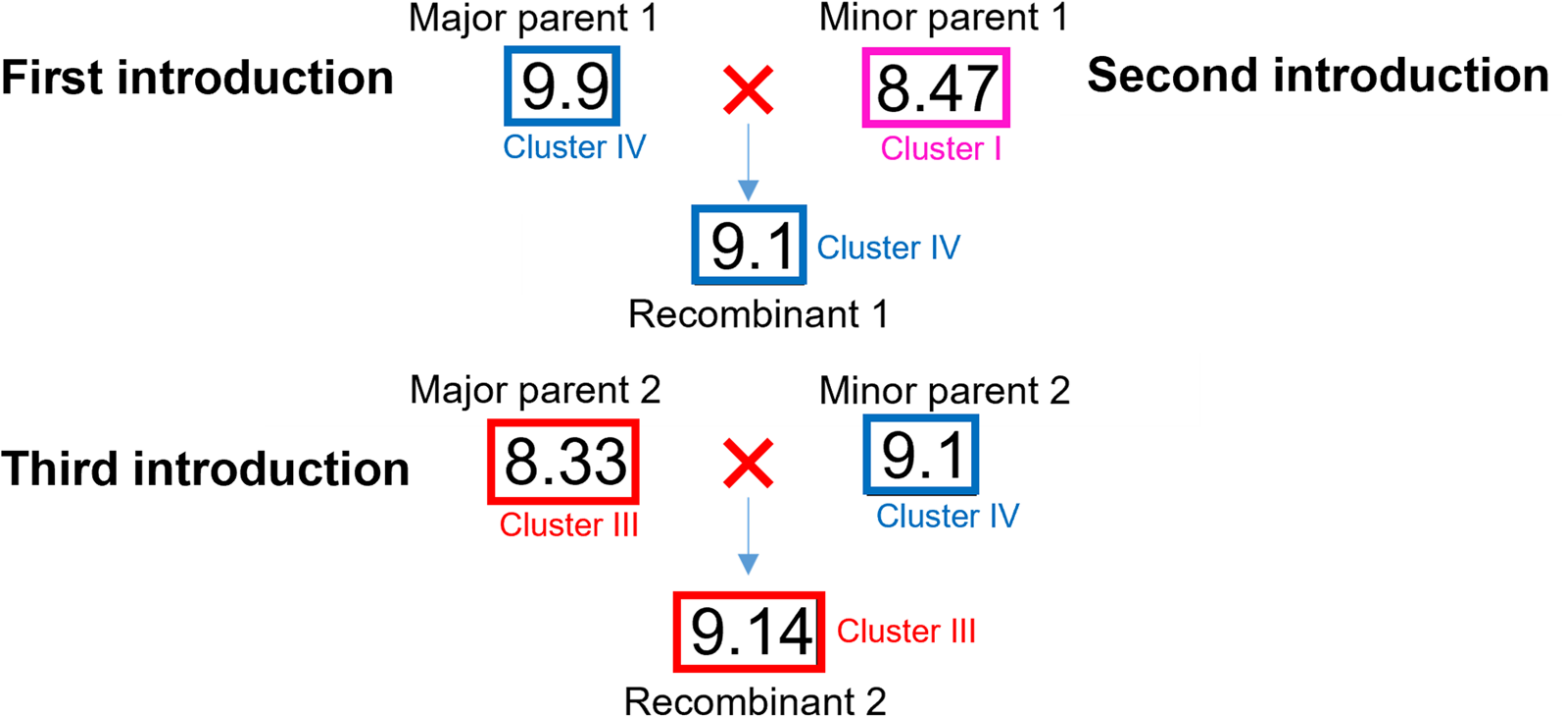
Hypothetical introductions of grapevine Pinot gris virus (GPGV) in Australia including the major and minor parents of predicted GPGV recombinants based on phylogenetic, median-joining (MJ) network and recombination analyses

Recombination events have been previously reported in GPGV [4, 5, 19] and this study also predicted recombination amongst the Australian isolates within the RdRp and MP regions. No international isolates were identified as parents which suggests that the evolution of these GPGV isolates occurred after their introduction into Australia (Table 4). Based on the phylogenetic, variant and recombination analysis, it is hypothesized that isolate 9.9 (major parent 1) could be an early introduction of GPGV into Australia which led to the generation of a recombinant 9.1 (recombinant 1) by combining with a second introduction of GPGV, 8.47 (minor parent 1). A second recombination event led to the formation of 9.14 (recombinant 2) which was formed by a recombination event between isolate 8.33 (major parent 2) and the recombinant isolate 9.1 (minor parent 2, Fig. 8). The prediction of Australian recombinants with Australian parent GPGV strains provides further evidence for the emergence of new variants since the initial introduction into Australia and could indicate that GPGV has been present here for a long time although the year of introduction could not be established.

The results of this study demonstrate that GPGV spread and divergence has occurred in Australia. Infected planting material is likely to have contributed to some spread within and between Australian table and wine grape growing regions. However, GPGV is also transmitted by the grape leaf bud-blister mites which has led to greater GPGV transmission efficiency [50]. The mite is dispersed through wind, transported through the movement of infested leaf materials, on clothing and equipment, and may be dispersed in cuttings [51]. The evidence for mite transmission lies in the diversity of *Vitis* species and varieties that are infected by GPGV, and which are represented in each phylogenetic clade and MJ network cluster. The close relatedness of the GPGV variants found in rootstocks, table grapes and wine grapes for example, isolate CK1 (rootstock), 8.28 (table grape) and 8.29 (wine grape) in phylogenetic clade II, which have > 99% nt identity, supports the hypothesis of localized transmission. A high abundance of blister mites has been observed in the Sunraysia horticultural district of VIC and NSW (data not shown) and would account for some of the spread and high prevalence of GPGV in this region. The lower prevalence of GPGV in other grape-growing regions in Australia might be associated with a lower abundance of the mite vector and possibly fewer introductions of the virus in planting material. There is evidence for the spread of GPGV between regions. For example, GPGV isolates in two wine grapes vines (unknown variety) in Angaston, SA, and an isolate in a table grape var. Ralli seedless from Sunraysia, VIC share > 99% nucleotide identity. The two regions are > 300km apart and this spread could be solely due to the movement of a viruliferous vector, but transmission between regions could also be due to the movement of infected planting material.

GLMD symptoms relating to GPGV infection are known to become visible during the beginning of the grapevine growing season, in spring, but fade later in the season towards veraison, when berries ripen [2]. It has been reported that there are mild and severe strains of GPGV that affect the severity of symptom expression [15]. Expression of GLMD symptoms in GPGV-affected vines collected as part of this study was variable with both symptomatic and asymptomatic vines of the same grape cultivar testing positive for the virus. There appeared to be no trend observed between cultivar, time of sampling (seasonality) or growing region and the expression of GLMD symptoms. These results were supported by the phylogenetic analyses conducted in this study. No association could be made between the presence of specific Australian GPGV strains and the presence of any symptom type in Australian grapevines when the same region was compared phylogenetically. These findings are in contrast to previous studies suggesting that two distinct genetic GPGV lineages represent asymptomatic and symptomatic grapevines [2, 6]. We observed strains of GPGV normally associated with asymptomatic infections overseas in grapevines with GLMD-like symptoms in Australia, and we have seen GPGV strains that would normally be associated with symptomatic vines in Italy in asymptomatic Australian grapevines. A similar lack of association between GPGV variants and disease has also been reported by others [52–54].

The correlation between symptoms associated with Australian GPGV isolates is further complicated by the presence of other viruses in some of the grapevines that were examined, which could also contribute to the presence of the disease (Additional file 1: Table S3). Similar results have been noted in other studies that reported the inability to link specific symptoms to the presence of GPGV given the mixed viral infection in grapevines [4, 55, 56].

GLMD-like symptoms, restricted spring growth, zig-zag shoots and millerandage, which were observed in some GPGV-infected grapevines in this study, can be caused by a range of abiotic and biotic factors such as other pathogens, environmental conditions, temperature, nutrient deficiency, soil type and presence of mites. It is possible these factors have contributed to the expression of these symptoms in some Australian grapevines that are infected with GPGV only or in combination with other viruses [2, 22, 57–59]. Variable GLMD expression has also been linked to boron deficiency [24]. Although the availability of boron was not measured in the Australian vineyards where GPGV-infected samples were collected for this study, soils in the Sunraysia region may be alkaline and with low organic matter which can affect boron content and availability and therefore may explain the variable association between GPGV presence and GLMD-like symptoms that were observed [60–64].

## Conclusions

In this study, a combination of the documented history of importation of some infected grapevine varieties into Australia, together with data obtained from surveillance and phylogenetic, MJ network, sequence identity and recombination analyses contributes to an improved understanding of GPGV introduction and spread in Australia. It indicated a minimum of five introductions of GPGV followed by the emergence of new variants. A high level of GPGV distribution in south-eastern Australian vineyards, particularly in Sunraysia, that was observed is likely linked to transmission by a bud-blister mite and distribution of infected planting material as has been observed in Italy [2, 5, 22]. Therefore in Australia, to minimize risk to production when establishing new vineyards, the use of planting material in which GPGV has not been detected is recommended and effective management of the bud mite vectors is required. The absence of a clear correlation between the distinct GPGV strains and the manifestation of symptoms in grapevines makes it necessary to conduct further research aimed at studying the biology of GPGV and its interaction with rootstocks, wine and table grape varieties grown in Australia.

### Supplementary Information

**Additional file 1**. **Figure S1**. Percentage identity for thirty-two genome sequences of grapevine Pinot gris virus (GPGV) which were de novo assembled from high throughput sequencing (HTS) datasets. **Figure S2**. Maximum likelihood tree with 1000 bootstrap replicates inferred from amino acid sequences of the protein-encoding Open reading frames (ORFs) of the thirty-two genome sequences of grapevine Pinot gris virus (GPGV) datasets. The scale bar corresponds to the number of substitutions per site. (a) RNA-dependent RNA polymerase protein (RdRp, ORF1) (b) Coat protein (CP, ORF3). The scale bar corresponds to the number of substitutions per site. Bootstrap values (> 70%) are reported at the nodes. **Table S1**: The primer pairs and their annealing temperatures used in endpoint RT-PCR and Sanger Sequencing to confirm the genome sequences of Australian grapevine Pinot gris virus (GPGV) isolates that were generated by high throughput sequencing (HTS). **Table S2**: The GenBank accession numbers for 168 GPGV genome sequences available on NCBI and their respective country of origin. **Table S3**: The sample identity, RT-PCR results, number of raw reads generated from metagenomic high-throughput sequencing (HTS), number of reads after quality trimming, number of contigs generated by SPAdes, number of viral contigs, number of grapevine Pinot gris virus (GPGV) contigs, GPGV contig size, percentage similarity to reference genome NC_015782, depth and mapped reads calculated using Geneious in each grapevine sample sequenced.

## Data Availability

The genomic data generated and/or analyzed during the current study are available in the open access database GenBank in National Center for Biotechnology Information repository (Accession numbers: OQ198990—OQ199021).
