# Supplementary material for: The genetic variability of grapevine Pinot gris virus (GPGV) in Australia
Source: Virol J. 2023 Sep 13;20:211. doi: 10.1186/s12985-023-02171-3 (PMC10500770; doi:10.1186/s12985-023-02171-3)
Supplement: Supplementary file 1 — Additional file 1. Figure S1. Percentage identity for thirty-two genome sequences of grapevine Pinot gris virus (GPGV) which were de novo assembled from high throughput sequencing (HTS) datasets. Figure S2. Maximum likelihood tree with 1000 bootstrap replicates inferred from amino acid sequences of the protein-encoding Open reading frames (ORFs) of the thirty-two genome sequences of grapevine Pinot gris virus (GPGV) datasets. The scale bar corresponds to the number of substitutions per site. (a) RNA-dependent RNA polymerase protein (RdRp, ORF1) (b) Coat protein (CP, ORF3). The scale bar corresponds to the number of substitutions per site. Bootstrap values (> 70%) are reported at the nodes. Table S1: The primer pairs and their annealing temperatures used in endpoint RT-PCR and Sanger Sequencing to confirm the genome sequences of Australian grapevine Pinot gris virus (GPGV) isolates that were generated by high throughput sequencing (HTS). Table S2: The GenBank accession numbers for 168 GPGV genome sequences available on NCBI and their respective country of origin. Table S3: The sample identity, RT-PCR results, number of raw reads generated from metagenomic high-throughput sequencing (HTS), number of reads after quality trimming, number of contigs generated by SPAdes, number of viral contigs, number of grapevine Pinot gris virus (GPGV) contigs, GPGV contig size, percentage similarity to reference genome NC_015782, depth and mapped reads calculated using Geneious in each grapevine sample sequenced. [file 12985_2023_2171_MOESM1_ESM.docx]

**Supplementary Tables**

#### Table S1. The primer pairs and their annealing temperatures used in endpoint RT-PCR and Sanger sequencing to confirm the genome sequences of Australian grapevine Pinot gris virus (GPGV) isolates that were generated by high throughput sequencing (HTS).

| **FORWARD primer name** | **FORWARD primer sequence (5’-3’)** | **Position**^1^ | **REVERSE primer name** | **REVERSE primer sequence (5’-3’)** | **Position^1^** | **Annealing Temp (°C)** | **Amplicon size** | **Reference** |
| --- | --- | --- | --- | --- | --- | --- | --- | --- |
| **GPG-14F** | AATTGATCCCGTGTAGTGC | 22 | **GPG-632R** | TCCGAGGACGATGAACCTC | 642 | 56 | 620 | Glasa et al 2014 |
| **GPGV_F9** | GATGAGGTATGGGATGGATG | 513 | **GPGV_R9** | TGGAGACTTACAGAATAATGCCC | 866 | 58 | 293 | This paper |
| **GPGV_F10** | CAGACCCTTGAGTCCTCATAT | 790 | **GPGV_R10** | GCTTGTTGGTATGATGTTGG | 1496 | 56 | 706 | This paper |
| **GPGV_F11** | ATTTGAGCCTGAGGAGGA | 1347 | **GPGV_R11** | GCTTGACGTTGACAGATTC | 1954 | 56 | 607 | This paper |
| **GPGV_F12** | CTGAAGGCTTCTTTGATCC | 1772 | **GPGV_R12** | GTGGTTTGAGACCCTCGAGAA | 2167 | 58 | 395 | This paper |
| **GPGV_F13** | CAGATTGAATCATCAGGTGA | 2028 | **GPGV_R13** | TTGATTGCAAGGTCTTCCACC | 2671 | 60 | 643 | This paper |
| **GPGV_F16** | CACCATGGTCTTCGTTTTC | 2587 | **GPGV_R16** | TTTGTCTCTCCAGTCTTCTG | 3238 | 54 | 652 | This paper |
| **GPGV_F17** | AAAGGGAAAGGGGTTGAGTC | 2878 | **GPGV_R17** | ACCATTCGGGAAAAGAGT | 3340 | 56 | 463 | This paper |
| **GPGV_F18** | TCGGGTTCAAATGAGATACT | 3173 | **GPGV_R18** | ATATGATTATCGGAACACAACC | 3804 | 56 | 631 | This paper |
| **GPGV_F6** | ACCTCAAGCACATTACACTC | 3594 | **GPGV_R6** | GCCAAGAAAGTCATCTCATC | 4545 | 55 | 952 | This paper |
| **GPGV_F14** | GAATTTCAGTGCTGTTTATCC | 4382 | **GPGV_R14** | CATCACCAGCAAAACAAACG | 5196 | 60 | 814 | This paper |
| **GPGVRepF** | TGAGGCATTCGATGTTTCCCA | 4940 | **GPGVRepR** | ACCCAATCAAGCCATGAACCT | 5461 | 60 | 521 | Saldarelli et al 2015 |
| **GPGV_F8** | GGCAGACCTCAAAGAATCAG | 5317 | **GPGV_R8** | CCTCACAGAAATGGCATAAC | 6218 | 54 | 902 | This paper |
| **GPGV_F19** | CCACGTGTTTATCCACCG | 6038 | **GPGV_R19** | TTCTGTGTAACCTGACATC | 6364 | 60 | 326 | This paper |
| **DetF** | TGGTCTGCAGCCAGGGGACA | 6225 | **DetR** | TCACGACCGGCAGGGAAGGA | 6812 | 60 | 700 | Morelli et al 2014 |

^1^ Position equivalent the genome sequence of the GPGV reference isolate NC_015782

#### Table S2. The GenBank accession numbers for 168 GPGV genome sequences available on NCBI and their respective country of origin.

| **Accession no.** | **Country** | **Accession no.** | **Country** | **Accession no.** | **Country** | **Accession no.** | **Country** | **Accession no.** | **Country** | **Accession no.** | **Country** | **Accession no.** | **Country** |
| --- | --- | --- | --- | --- | --- | --- | --- | --- | --- | --- | --- | --- | --- |
| BK011060 | Italy | BK011085 | China | BK011109 | Uruguay | MH087439 | Italy | MN458411 | France | MN458435 | France | MN458459 | France |
| BK011061 | Italy | BK011086 | China | FR877530 | Italy | MH087440 | Italy | MN458412 | France | MN458436 | France | MN458460 | France |
| BK011062 | Italy | BK011087 | China | KF134123 | Slovak and Czech | MH087441 | Italy | MN458413 | France | MN458437 | France | MT414711 | Greece |
| BK011063 | Italy | BK011088 | China | KF134124 | Slovak and Czech | MH087442 | Italy | MN458414 | France | MN458438 | France | MW815134 | Greece |
| BK011064 | Italy | BK011089 | Italy | KF134125 | Slovak and Czech | MH087443 | Italy | MN458415 | France | MN458439 | France | MZ220969 | British Columbia |
| BK011065 | Italy | BK011090 | Italy | KF686810 | Slovak and Czech | MH087444 | Italy | MN458416 | France | MN458440 | France | MZ220970 | British Columbia |
| BK011066 | Italy | BK011091 | Italy | KM491305 | France | MH087445 | Italy | MN458417 | France | MN458441 | France | MZ344578 | British Columbia |
| BK011067 | Italy | BK011092 | Italy | KP693444 | Czech Republic | MH087446 | Italy | MN458418 | France | MN458442 | France | MZ344580 | British Columbia |
| BK011068 | Italy | BK011093 | Italy | KP693445 | Czech Republic | MH087447 | Italy | MN458419 | France | MN458443 | France | NC_015782 | Italy |
| BK011069 | Italy | BK011094 | Italy | KP693446 | Czech Republic | MH802023 | British Columbia | MN458420 | France | MN458444 | France | OL961512 | Russia |
| BK011070 | Italy | BK011095 | Italy | KP693447 | Czech Republic | MK514520 | California | MN458421 | France | MN458445 | France | ON237610 | Switzerland |
| BK011071 | Italy | BK011096 | Italy | KP693448 | Czech Republic | MK514521 | California | MN458422 | France | MN458446 | France | ON548147 | Russia |
| BK011072 | Italy | BK011097 | Italy | KR528581 | Korea | MK514522 | California | MN458423 | France | MN458447 | France | ON548148 | Russia |
| BK011073 | China | BK011098 | Italy | KT894101 | California | MK514523 | California | MN458424 | France | MN458448 | France | ON548149 | Russia |
| BK011074 | China | BK011099 | Italy | KU194413 | British Columbia | MK514524 | California | MN458425 | France | MN458449 | France | ON548150 | Russia |
| BK011075 | China | BK011100 | Italy | KU312039 | Italy | MK514525 | California | MN458426 | France | MN458450 | France | ON548151 | Russia |
| BK011076 | China | BK011101 | Italy | KU508673 | China | MK514526 | California | MN458427 | France | MN458451 | France | ON548152 | Russia |
| BK011078 | Italy | BK011102 | France | KU949328 | Slovakia | MK514527 | California | MN458428 | France | MN458452 | France | ON548153 | Russia |
| BK011079 | Italy | BK011103 | France | KX522755 | Germany | MK514528 | California | MN458429 | France | MN458453 | France | ON567239 | Russia |
| BK011080 | British Columbia | BK011104 | China | KY706085 | France | MK514531 | California | MN458430 | France | MN458454 | France | ON620246 | Russia |
| BK011081 | Italy | BK011105 | China | KY747493 | Pakistan | MK514532 | California | MN458431 | France | MN458455 | France | ON620247 | Russia |
| BK011082 | China | BK011106 | China | KY747494 | Pakistan | MK514533 | California | MN458432 | France | MN458456 | France | ON620248 | Russia |
| BK011083 | China | BK011107 | China | LC601811 | Japan | MN093125 | France | MN458433 | France | MN458457 | France | ON620249 | Russia |
| BK011084 | China | BK011108 | Uruguay | LC601812 | Japan | MN228488 | Belgium | MN458434 | France | MN458458 | France | ON620250 | Russia |

#### Table S3. The sample identity, RT-PCR results, number of raw reads generated from metagenomic high-throughput sequencing (HTS), number of reads after quality trimming, number of contigs generated by SPAdes, number of viral contigs, number of grapevine Pinot gris virus (GPGV) contigs, GPGV contig size, its percentage similarity to reference genome NC_015782.2, depth and mapped reads calculated using Geneious in each grapevine sample sequenced.

| **Sample Identity** | **RT-PCR** | **RT-qPCR (Ct value)** | **Total number of raw reads** | **Reads after trimming (Fastp)** | **No of viral contigs** | **No of GPGV contigs** | **Assembled GPGV contig size (nt)** | **Percentage similarity to Reference genome NC_015782.2 (at the end)** | **Depth (Geneious)** | **Mapped reads (Geneious)** | **Other viruses** |
| --- | --- | --- | --- | --- | --- | --- | --- | --- | --- | --- | --- |
| CK1 | NA | NA | NA | NA | NA | NA | NA | NA | NA | NA | NA |
| 2.1 | + | 20.1 | 30198881 | 29489728 | 29 | 6 | 7183 | 99.42% | 14 | 699 | RSPaV |
| 2.12 | + | 25.6 | 1399151 | 1387616 | 39 | 3 | 7251 | 97.96% | 19 | 903 | RSPaV |
| 2.17 | + | 25.7 | 2532276 | 2515923 | 145 | 5 | 6700 | 98.41% | 89 | 4300 | GLRaV 1, GLRaV 3, GVA, RSPaV |
| 5.5^b^ | + | 22.9 | 1572989 | 1566231 | 18 | 7 | 7162 | 98.24% | 7 | 320 | RSPaV |
| 5.6 | + | 24.9 | 11935852 | 11908338 | 127 | 1 | 7265 | 98.17% | 8 | 411 | RSPaV, LR3, GVA |
| 5.13^b^ | + | 24.1 | 3367102 | 3352925 | 1197 | 3 | 7047 | 98.24% | 16 | 756 | RSPaV, LR3 |
| 5.14 | + | 23.9 | 6607729 | 6590758 | 755 | 1 | 7426 | 98.34% | 16 | 756 | RSPaV, GVA |
| 5.17 | + | 23.4 | 3148666 | 3133701 | 23 | 2 | 7338 | 98.24% | 11 | 516 | RSPaV |
| 5.21^a^ | + | 23.5 | 2020590 | 2011880 | 31 | 3 | 7449 | 98.84% | 5 | 227 | RSPaV, LR3 |
| 5.22 | + | 20.3 | 2436767 | 2427356 | 16 | 2 | 7381 | 98.33% | 22 | 1062 | RSPaV, LR3 |
| 5.24^b^ | + | 21.8 | 1719154 | 1711195 | 42 | 2 | 7288 | 97.73% | 7 | 331 | RSPaV |
| 8.6 | + | 31 | 10976041 | 10945015 | 2674 | 12 | 7286 | 97.55% | 53 | 2578 | RSPaV |
| 8.7 | + | 31.4 | 2181942 | 2176512 | 45 | 1 | 7357 | 98.28% | 40 | 1917 | None |
| 8.28 | + | 29.2 | 11919689 | 11879972 | 179 | 1 | 7249 | 98.49% | 25 | 1192 | RSPaV, GFkV, |
| 8.29 | + | 31.2 | 5601426 | 5584651 | 17 | 3 | 7267 | 98.43% | 10 | 482 | RSPaV, GVA |
| 8.33 | + | 33 | 2422424 | 2413789 | 53 | 2 | 7267 | 98.74% | 5 | 246 | RSPaV |
| 8.38 | + | 23.3 | 5027466 | 5011921 | 57 | 1 | 7490 | 98.31% | 30 | 1432 | RSPaV |
| 8.47 | + | 29.4 | 10909383 | 10875757 | 63 | 1 | 7072 | 98.26% | 13 | 611 | RSPaV |
| LT6^a^ | + | 19.8 | 2255212 | 2243583 | 28 | 3 | 7177 | 98.14% | 10 | 498 | RSPaV |
| LT7^b^ | + | 24 | 3858481 | 3842999 | 125 | 5 | 6990 | 98% | 5 | 218 | RSPaV |
| 9.1 | + | 22 | 19352457 | 19305864 | 47 | 9 | 7227 | 97.48% | 80 | 3861 | RSPaV |
| 9.2 | + | 21 | 12910078 | 12878157 | 38 | 1 | 7274 | 98.45% | 32 | 1567 | RSPaV |
| 9.3 | + | 22.1 | 32193380 | 32121154 | 29 | 1 | 7319 | 98.31% | 84 | 4044 | LR3 |
| 9.4 | + | 22.3 | 11502466 | 11476928 | 23 | 2 | 7329 | 98.26% | 33 | 1600 | None |
| 9.5 | + | 21.8 | 9996248 | 9974732 | 62 | 1 | 7270 | 98.20% | 76 | 3672 | GFkV, LR1, GVA |
| 9.6 | + | 20.9 | 15475505 | 15438113 | 19 | 1 | 7329 | 98.40% | 117 | 5680 | RSPaV |
| 9.9 | + | 21.2 | 9601270 | 9581148 | 52 | 1 | 7231 | 97.95% | 70 | 3391 | RSPaV |
| 9.1 | + | 21.7 | 21710874 | 21650287 | 87 | 5 | 7274 | 98.27% | 45 | 2157 | RSPaV |
| 9.11 | + | 22 | 11337043 | 11314547 | 75 | 2 | 7280 | 98.45% | 23 | 1092 | RSPaV |
| 9.13 | + | 20.3 | 10604062 | 10580901 | 74 | 6 | 7366 | 98.09% | 123 | 5946 | GFkV |
| 9.14 | + | 23.6 | 9760651 | 9743467 | 46 | 7 | 7284 | 98.34% | 120 | 5819 | GFkV |

* a- sequences confirmed with Sanger sequencing

* b- sequences with gaps and low coverage areas confirmed with Sanger sequencing

**Supplementary Figures**


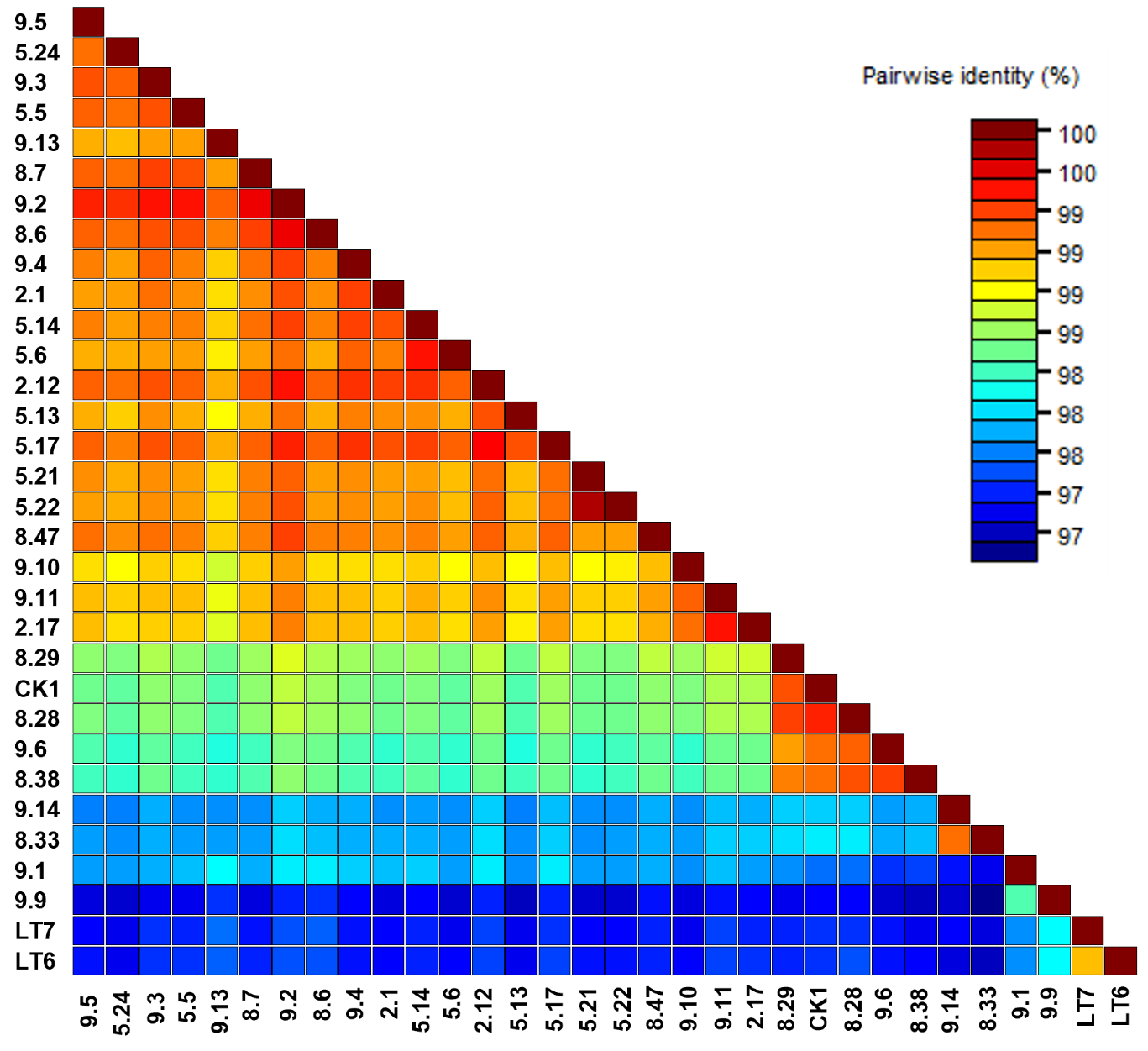


#### Figure S1. Percentage identity for thirty-two sequences of Grapevine pinot gris virus (GPGV) which were de novo assembled from high throughput sequencing (HTS) datasets.


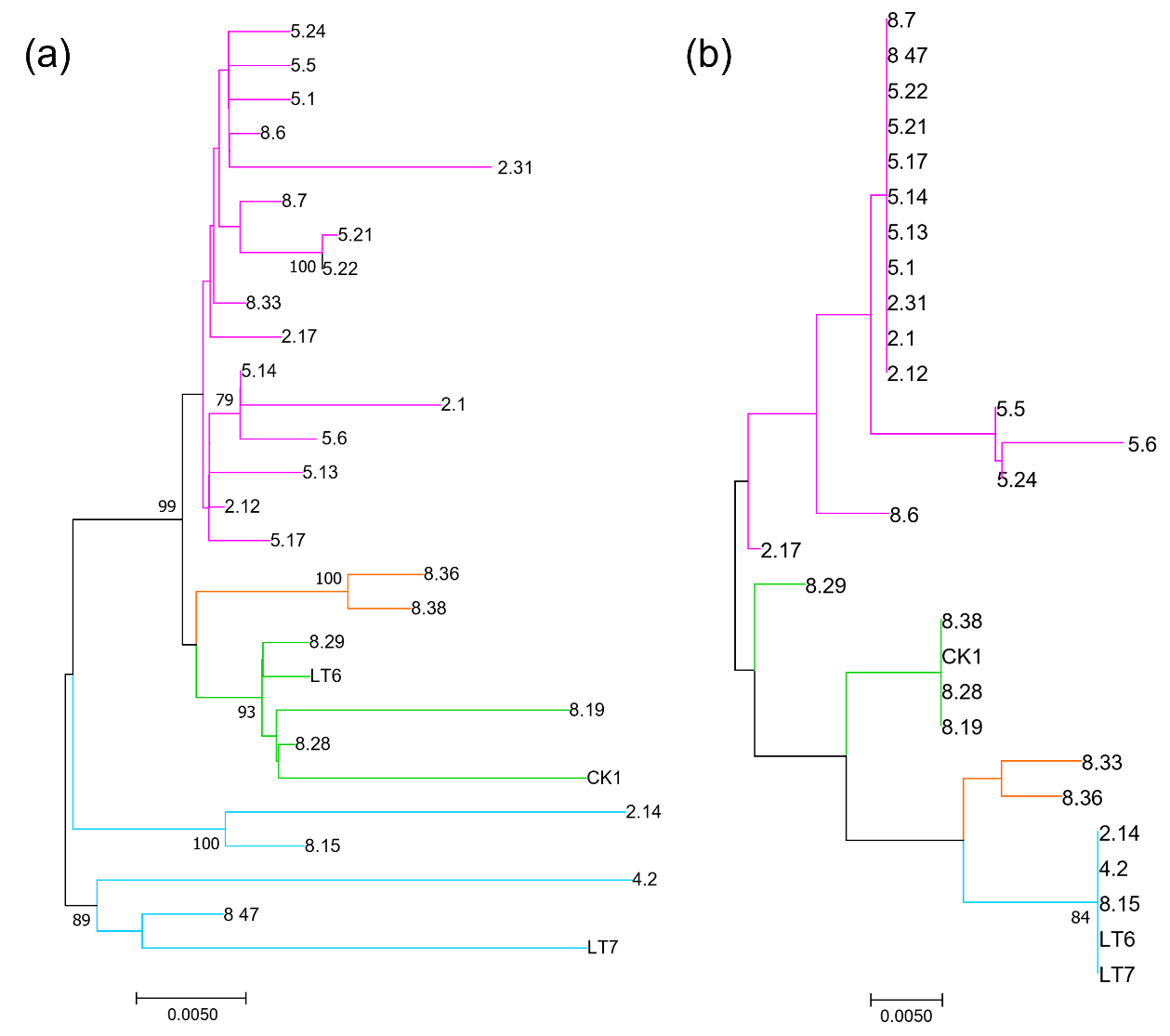


#### Figure S2. Maximum likelihood tree with 1000 bootstrap replicates inferred from amino acid sequences of the protein encoding Open reading frames (ORFs) of the thirty-two sequences of grapevine Pinot gris virus (GPGV) datasets. The scale bar corresponds to the number of substitutions per site. (a) RNA-dependent RNA polymerase protein (RdRp, ORF1) (b) Coat protein (CP, ORF3). The scale bar corresponds to the number of substitutions per site. Bootstrap values (>70%) are reported at the nodes.
